# The influence of habitat structure on genetic differentiation in red fox populations in north-eastern Poland

**DOI:** 10.1007/s13364-014-0180-2

**Published:** 2014-03-22

**Authors:** Jacinta Mullins, Allan D. McDevitt, Rafał Kowalczyk, Iwona Ruczyńska, Marcin Górny, Jan M. Wójcik

**Affiliations:** 1 Mammal Research Institute, Polish Academy of Sciences, 17-230 Białowieża, Poland; 2 School of Biology and Environmental Science, University College Dublin, Belfield, Dublin 4, Ireland

**Keywords:** Microsatellites, Spatial autocorrelation, Bayesian clustering, Landscape resistance, Least-cost path

## Abstract

The red fox (*Vulpes vulpes*) has the widest global distribution among terrestrial carnivore species, occupying most of the Northern Hemisphere in its native range. Because it carries diseases that can be transmitted to humans and domestic animals, it is important to gather information about their movements and dispersal in their natural habitat but it is difficult to do so at a broad scale with trapping and telemetry. In this study, we have described the genetic diversity and structure of red fox populations in six areas of north-eastern Poland, based on samples collected from 2002–2003. We tested 22 microsatellite loci isolated from the dog and the red fox genome to select a panel of nine polymorphic loci suitable for this study. Genetic differentiation between the six studied populations was low to moderate and analysis in Structure revealed a panmictic population in the region. Spatial autocorrelation among all individuals showed a pattern of decreasing relatedness with increasing distance and this was not significantly negative until 93 km, indicating a pattern of isolation-by-distance over a large area. However, there was no correlation between genetic distance and either Euclidean distance or least-cost path distance at the population level. There was a significant relationship between genetic distance and the proportion of large forests and water along the Euclidean distances. These types of habitats may influence dispersal paths taken by red foxes, which is useful information in terms of wildlife disease management.

**Electronic supplementary material:**

The online version of this article (doi:10.1007/s13364-014-0180-2) contains supplementary material, which is available to authorized users.

## Introduction

Gene flow between populations or groups of animals is dictated by a multitude of internal (i.e., vagility/dispersal) and external (i.e., landscape and environmental features) factors (Pérez-Espona et al. 2008). It has been well established that landscape features affect the dispersal ability of animals and therefore impact upon gene flow and the genetic structure of populations (Manel et al. 2003; Sommer et al. 2013). Larger terrestrial mammals with higher dispersal capabilities may be expected to be less perturbed by geographic distances but may be more limited by the landscape matrix separating putative populations. An understanding of the potential limiting factors on the landscape for movement and gene flow between populations is important for conservation purposes (Sommer et al. 2013) but is also particularly relevant for widespread species capable of spreading disease to both humans and animals closely associated with them (Biek and Real 2010). Studies using landscape resistance models have often lacked empirical data and instead relied heavily upon expert opinion to identify habitat variables important to resistance (Spear et al. 2010). In recent years, landscape genetic studies have begun to incorporate telemetry data from a variety of fitted collars (radio, GPS, etc.) that more accurately reflect dispersal and movement across the landscape (McDevitt et al. 2013) and aid in identifying key environmental metrics for constructing objective landscape resistance surfaces (Shafer et al. 2012; Weckworth et al. 2013).

The red fox *Vulpes vulpes* has the largest contemporary range of any carnivore species, with a native range that covers most of the temperate and subarctic regions of the Northern Hemisphere (MacDonald and Reynolds 2008). Population expansion and the colonization of several European towns and cities in the last century has prompted concerns about the transmission of parasitic and viral diseases such as rabies and the fox tapeworm *Echinococcus multilocularis* to humans and companion animals (Chautan et al. 2000; Deplazes et al. 2004). Studies of the genetic structure of fox populations have yielded valuable insights into the behavioral and spatial ecology of foxes (Wandeler et al. 2003; De Young et al 2009). Wandeler et al. (2003) analyzed patterns of differentiation at microsatellites between fox populations in and around the city of Zürich, Switzerland. The highest level of differentiation was found between the urban populations either side of a river, but there was evidence of gene flow between urban and rural populations. Several studies using microsatellites and mitochondrial DNA (Oishi et al. 2010; Magory Cohen et al. 2012; Galov et al. 2014) described the genetic structure among red fox populations over wide geographical areas and revealed a pattern of weak genetic structure, highlighting the adaptability and dispersal potential of red foxes.

In Poland, the red fox is a common predator with the highest population growth of all carnivores in Polish national parks (Jamrozy 2008), despite periods of severe population exploitation and epizootic disease in the last century (Jędrzejewska and Jędrzejewski 1998). Our objective in this study was to describe the overall genetic structure of red fox populations and the factors affecting landscape connectivity in north-eastern Poland, a semi-natural landscape with high forest cover. We expected high rates of dispersal and weak genetic structure given the dispersal capabilities of the species and hypothesized that large continuous forests may be avoided during dispersal as foxes prefer to forage in mosaic or open habitats where their preferred prey, microtine voles, are more abundant (Jędrzejewska and Jędrzejewski 1998; Kidawa and Kowalczyk 2011).

## Material and methods

### Study area and sample collection

**Fig. 1:**
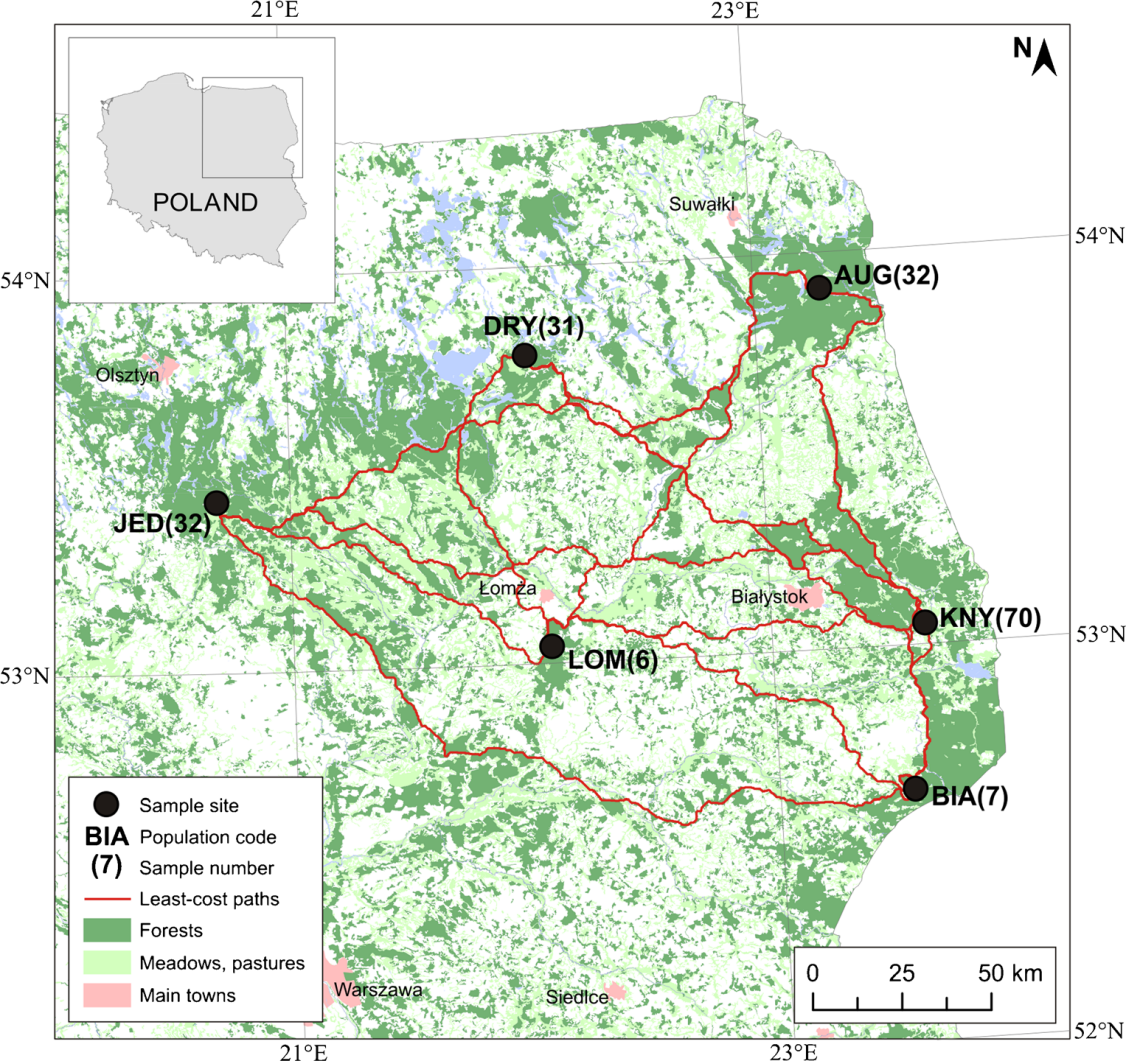
Study area in north-eastern Poland, with sampling locations and the least-cost paths between each location

In this study, we investigated six populations of the red fox in north-eastern Poland (Fig. 1). Well-preserved natural environments characterize this region. Predominant habitats in the study area are forests, grasslands, wastelands, and wetlands (for details see Kidawa and Kowalczyk 2011) and sampling reflected these wide arrays of habitat types. Augustów Forest is an extensive forest dominated by coniferous (85 % of the forest area) and alder bog forests. Drygały and Jedwabno are old military training grounds primarily covered in coniferous forest and wetland habitats. The Łomża area is also a reforested military training ground, dominated by pine forest, within which many open stands occur. Białowieża and Knyszyn Forests are more heterogeneous areas covered by deciduous, mixed, and coniferous forest (42 % of the area) and less intensively cultivated agricultural fields (meadows, pastures, wastelands). In many places, abandoned fields are encroached by different stages of forest succession.

Kidawa and Kowalczyk (2011) described the diet of the red fox based on the stomach content of 224 individuals shot as part of control operations in north-eastern Poland between September 2002 and March 2003. In this study, we used muscle tissue samples from 178 foxes, representing six sampling locations (Fig. 1): Augustów E 23° 28′ N 53° 91′ (AUG, *N* = 32), Białowieża E 23° 56′ N 52° 63′ (BIA, *N* = 7), Drygały E 22° 02′ N 53° 78′ (DRY, *N* = 31), Jedwabno E 20° 69′ N 53° 43′ (JED, *N* = 32), Knyszyn E 23° 64′ N 53° 05′ (KNY, *N* = 70), and Łomża E 22° 08′ N 53° 04′ (LOM, *N* = 6). The approximate latitude and longitude geographic coordinates of capture were available for all individuals.

### Genotyping

Genomic DNA was extracted from all samples using the QIAGEN DNeasy Tissue Kit, according to the standard protocol for animal tissue. We tested 15 published canine microsatellite loci: FH2001, FH2010, FH2054, FH2079, FH2088, FH2096, FH2137, and FH2140 (Francisco et al. 1996); VWF (Shibuya et al. 1994); C213, C250, C253, C466, C642 (Ostrander et al. 1993); and AHT130 (Holmes et al. 1995), and designed primers for an additional 27 loci (GenBank Accession numbers: JN831722-JN831749; Yan, unpublished) using WebSat (Martins et al. 2009). These loci were isolated from the genome of a silver fox, which is a domesticated form of the North American lineage of the red fox (Statham et al. 2011). Seven of these additional loci were chosen (VVM33, VVM39, VVM63, VVM81, VVM189, VVM124, and VVM828) and the forward primer was labeled with either VIC, 6-FAM, or PET fluorescent dye for fragment analysis. The primers and additional information are given in Table S1. In total, 22 loci were tested (15 published and seven additional loci). We retained loci based on ease of amplification, polymorphism, ease of scoring, low frequency of null alleles (<0.1) and conformance to Hardy-Weinberg equilibrium (HWE) from an initial pilot study of 23 adult foxes from Augustów Forest. We used the software Micro-Checker 2.3.3 (van Oosterhout et al. 2004) and HW-QuickCheck (Kalinowski 2006) to calculate null allele frequencies and carry out the HWE tests.

The PCR conditions for the final set of nine loci (see Results) were as follows: loci C250 and FH2096 were amplified separately in 10-μL volumes with 0.25-U Maxima HS Taq DNA polymerase (Fermentas), 1X PCR buffer, 0.2-mM dNTP, 2-mM MgCl2, 0.2-μM (C250), or 0.4-μM (FH2096) of each primer and 1 μL of genomic DNA extract (approximately 50 ng). These loci were amplified using a touchdown protocol of 95 °C for 4 min, followed by 7 cycles of 95 °C for 30 s, 63 °C for 30 s, and 72 °C for 30 s with a second step of 27 cycles of 95 °C for 30 s, 56 °C for 30 s, 72 °C for 30 s and final extension for 10 min at 72 °C. The remaining loci were amplified in 5-μL multiplexes of 2–3 loci per reaction, with 1X KAPA2G Fast PCR multiplex mastermix (Kapa Biosystems), 0.8 μM (FH2137), or 0.4 μM (all remaining loci) of each primer and 1 μL of genomic DNA extract. The multiplex PCR protocol was 95 °C for 3 min, followed by 30 cycles of 95 °C for 15 s, 60 °C for 30 s, 72 °C for 30 s, with a final extension for 10 min at 72 °C. All DNA extractions were carried out in a separate lab with dedicated equipment and both the extractions and PCRs were monitored for contamination using negative controls.

Fragment analysis was carried out with an ABI PRISM 3130xl Genetic Analyzer under standard run conditions with LIZ500 as the internal size standard. Alleles were scored and binned in GENEMARKER 1.7 (SoftGenetics), except for locus FH2054 that showed a compound repeat pattern, making it difficult to manually assign bins. We used the automated binning software Tandem (Matschiner and Salzburger 2009) for this locus, and rescored the alleles in GeneMarker using the output from this program.

### Data analysis

Genetic diversity was evaluated for the global sample using the number of alleles (*A*), observed heterozygosity (*H*_O_), and expected heterozygosity (*H*_E_) using the R package ADEGENET 1.3.6 (Jombart 2008). Values of allelic richness, observed and expected heterozygosity, and the inbreeding coefficient (*F*_IS_) for each population were calculated using FSTAT 2.9.3 (Goudet 2001) and Arlequin 3.5 (Excoffier and Lischer 2010). We used the exact test in GENEPOP 4.2 (Rousset 2008) to test for significant departures from HWE within each location, along with testing for evidence of linkage between loci using the G-test. The same Markov Chain parameters were used for both tests: 10,000 dememorizations, followed by 1,000 batches of 5,000 iterations per batch, with *p* values adjusted for multiple tests (Rice 1989).

We used the simulation program POWSIM 4.1 (Ryman and Palm 2006) to evaluate the statistical power of the final panel to detect genetic differentiation among the sampled populations. The global allele frequencies were used as a base population to create subpopulations of equal effective population size (Ne) through random sampling. Each of the subpopulations was then allowed to drift for 10, 25, 50, or 101 generations to give predefined *F*_ST_ values of 0.001, 0.0025, 0.005, and 0.01, respectively (Ne was set to 5,000). The null hypothesis of homogeneity in allele frequencies among all the subpopulations was tested with both the chi-squared test and the Fisher’s exact test (Markov Chain parameters: 10,000 burn-in; 100 batches; 1,000 iterations per batch) after the drift process. This process was repeated 1,000 times and the proportion of significant (*p* < 0.05) tests was used as an estimate of power. The type-I error was estimated with the same parameters but no generations of drift, to give an expected *F*_ST_ of zero.

We calculated pairwise *F*_ST_ (Weir and Cockerham 1984) and *D* (Jost 2008) between the sampled populations using the software GenoDive (Meirmans and van Tienderen 2004). Significance was assessed for pairwise differentiation with 9,999 permutations. Bayesian clustering methods are widely applied in population genetics studies to identify the number of genetic cluster(s) (*K*) that best fit the data by grouping individual genotypes such that HWE and linkage disequilibrium (LD) are minimized. We used Structure 2.3.4 (Pritchard et al. 2000), using the admixture model with correlated allele frequencies without prior population information. We carried out five replicates for 1 ≤ *K* ≤ 8 with 600,000 Markov Chain Monte Carlo (MCMC) iterations, discarding the first 100,000 as burn-in. We used Structure Harvester (Earl and Von Holdt 2011) to visualize the results and chose the optimal *K* based on the plot of the mean posterior log-likelihood for each *K*.

We used the spatial autocorrelation analysis (Smouse and Peakall 1999) in GenAlEx 6.5 (Peakall and Smouse 2012) to evaluate the null hypothesis of random genetic structure across the entire study area. The autocorrelation coefficient, *r*, was calculated for each pair of individuals and divided into 30 distance classes with approximately equal sample sizes in each distance class (mean *N* = 574.8 ± 87.2). These values are sufficient to achieve high statistical power (Epperson 2005). A null distribution of *r* values for each distance class was obtained by permutation (*N* = 9999) and the confidence intervals about *r* were estimated by bootstrapping with replacement (*N* = 9999). Significant spatial genetic structure was inferred when *r* exceeded the null distribution for a given distance class and the confidence intervals did not overlap zero. The results were plotted in a correlogram and the overall significance was evaluated with the heterogeneity test (Smouse et al. 2008). The distance class at which *r* is no longer significant and the x-intercept give an approximation of the extent of the detectable positive spatial genetic structure (Peakall et al. 2003). We carried out this analysis for both sexes combined and for each sex separately to assess differences between the sexes in dispersal behavior.

### Landscape connectivity

We explored two measures of spatial distance between populations: Euclidean distance and least-cost path (LCP) distance (Adriaensen et al. 2003; Verbeylen et al. 2003). The first model (Euclidean distance) represented a null hypothesis where all landscape features were assumed to be equally permeable for foxes. For the second model (LCP distance), we calculated effective distances between populations that take into account the effect of landscape features on fox movement. We performed both analyses in ArcGIS 9.3.1 software with Landscape Genetics Toolbox (Etherington 2011).

Euclidean distances are equal to straight-line geographic distances between each pair of populations. These distances were calculated in Euclidean distances tool from Landscape Genetics using Poland CS92 coordinates of each population. To perform LCP analyses, we created a habitat map for the study area. We used Corine Land Cover 2006 Project (CLC06) (European Environment Agency 1990–ongoing). The CLC06 classes were grouped into six land cover categories (Table 1). Next, this habitat map was transformed into a friction map, describing the resistance of each landscape categories to fox movement. The resistance values for each landscape feature was extracted from habitat selectivity analyses based on radio-tracking data from three collared individuals from Białowieża Forest (Kowalczyk et al., unpublished data) and results of habitat selection analysis from Finland (Holmala and Kauhala 2009). Habitat selectivity was calculated using Jacobs selectivity index (Jacobs 1974) widely used in habitat selection analysis. The index, which varies between +1 and −1, when +1 is the most suitable and −1 is the most unsuitable habitat, was then recalculated to a resistance values in a scale from 1 (the most suitable habitat) to 100 (the most unsuitable habitat; Klug et al. 2011). Results of habitat selection analyses for foxes shows that this carnivore generally prefer fragmented and diverse landscape and avoids large forest patches as well as large continuous arable fields (Holmala and Kauhala 2009). Therefore, we decided to split forest and arable land categories into two categories, respectively: forest edge (300-m zone on the edge of the forest), forest (forest habitats >300-m from its edge), arable land edges (300-m wide zone of fields from the forest edge), and arable land (fields located >300-m from the forest edge). The 300-m distance was selected on the basis of radio-tracking data from three collared individuals from Białowieża Forest (Kowalczyk et al., unpublished data). Resistance values of different habitats based on habitat selection by radio-collared foxes are presented in Table 1. The friction map was converted to raster grid with a resolution of 100 m using ArcGIS Polygon to Raster tool. The selected resolution corresponded with the given resolution of CLC06 data (described as 25-ha minimum area and 100-m minimum wide of the single habitat feature). Finally, the LCP algorithm (also called the Dijkstra algorithm (Dijkstra 1959)) was used to determine the shortest-cost path distances between populations. Then, in the final step, pairwise least-cost path distances were calculated between all the fox population in ArcGIS Landscape Genetics Least-Cost Path Tool.

**Table 1:** Resistance values of different habitats based on habitat selection by radio-collared foxes in Białowieża Forest and mean proportion of given habitats on Euclidian distances between examined red fox populations

Land cover categories	Corine land cover codes	Jacobs’ index	Resistance values	Mean proportion of given habitat along Euclidian distances (%)
Forest	311, 312, 313, 324	−0.13	32	23.4
Forest edge (300 m)	311, 312, 313, 324	0.28	1	18.3
Grasslands, wastelands, wetlands, and extensive agriculture	131, 132, 133, 231, 243, 411, 412	0.26	3	21.7
Villages and scattered settlements	141, 142, 222, 242	0.07	16	3.7
Urban development	111, 112, 121, 122, 123, 124	−0.26	42	1.7
Arable land	211	−0.20	38	7.1
Arable land edges (300 m)	211	0.23	5	22.1
Water	511, 512	−1.00	100	2.0

We used simple Mantel tests (Mantel 1967) to evaluate the relationship between genetic distance (*F*_ST_/1 − *F*_ST_; Rousset 1997) and the natural log of each geographic distance (Euclidean or LCP distance) in our study area. We used Mantel tests because they are easy to interpret, are widely used, are appropriate for distance data (Legendre and Fortin 2010) and are shown to correctly identify drivers of genetic diversity (Cushman and Landguth 2010; Weckworth et al. 2013). Mantel tests were carried out in ZT (Bonnet and Van de Peer 2002) with 10,000 permutations to assess significance. In addition, we also calculated the relationship between genetic distance (*F*_ST_/1 − *F*_ST_) and the proportion of given habitats along the Euclidean distances for the populations examined. When a significant relationship was observed between genetic distance and a particular habitat proportion, we also tested this given habitat in combination with each of the non-significant given habitats to test if this strengthened the relationship with genetic distance (see Results).

## Results

### Selection of microsatellites for population genetics of red foxes

Of the 22 microsatellites isolated from the dog and the red fox genome, nine loci (FH2001, FH2079, FH2140, AHT130, C213, C253, C642, VVM39, and VVM63) either failed to amplify or were difficult to score. VWF amplified reliably and was easily scored, but only one allele was detected among 23 individuals in the pilot study (Table S2). Three of the remaining 12 loci (FH2088, VVM81, and VVM33) did not conform to HWE (Table S2). Previous researchers have reported problems with FH2088 (Soulsbury et al. 2007) and preferential allele amplification was apparent for VVM81. We continued with nine loci for the remaining analyses (Table 2). The number of alleles per locus ranged from 3 at FH2096 to 22 at FH2054. The paired average observed heterozygosity (0.73) and expected heterozygosity (0.72) values were similar for each locus, with no HWE deviations in each location or overall. The results from the power simulations suggested that our sampling design with these loci should have sufficient power (>0.99) to detect low genetic differentiation (*F*_ST_ > 0.01) between populations (Figure S1).

**Table 2:** Characteristics of the final panel of microsatellite loci used in this study

Locus	Size range	A	*H*_*O*_	*H*_*E*_
FH2010	215–227	4	0.562	0.584
FH2054	143–203	22	0.860	0.880
FH2096	142–158	3	0.508	0.527
FH2137	148–188	11	0.831	0.818
C250	112–136	11	0.736	0.764
C466	135–153	8	0.747	0.760
VVM124	234–252	10	0.674	0.715
VVM189	227–249	12	0.843	0.865
VVM828	215–237	11	0.809	0.831

*A* number of alleles, *H*_*O*_ observed heterozygosity, *H*_E_ expected heterozygosity

### Genetic variability within and between populations and genetic structure

Allelic richness (*A*) ranged from 4.444 in Łomża (LOM) population to 5.281 in Białowieża (BIA) population (Table 3). Observed heterozygosity (*H*_O_) values were not significantly different from expected heterozygosity (*H*_E_) values in each population. Inbreeding coefficient (*F*_IS_) was greatest in the Białowieża population (0.071) and smallest in Jedwabno (JED) population (0.009) (Table 3). *F*_IS_ values were not significant for all populations (*p* = 0.143–0.408).

**Table 3:** Diversity indices for red fox populations examined

No.	Population	Code	*n*	*A*	*H*_O_	*H*_E_	*F*_IS_	*p*
1	Augustów	AUG	32	5.067	0.7465	0.7648	0.024	0.238
2	Białowieża	BIA	7	5.281	0.7460	0.7985	0.071	0.157
3	Drygały	DRY	31	4.777	0.7240	0.7416	0.024	0.289
4	Jedwabno	JED	32	4.737	0.7307	0.7376	0.009	0.386
5	Knyszyn	KNY	70	4.921	0.7254	0.7430	0.024	0.143
6	Łomża	LOM	6	4.444	0.7037	0.7222	0.028	0.408

*n* sample size; *A* allelic richness; *H*_O_ observed heterozygosity; *H*_E_ expected heterozygosity; *F*_IS_ inbreeding coefficient, and associated *p* value for *F*_IS_ based on 1,080 randomizations

Genetic differentiation between the six sample locations was low to moderate, with the highest pairwise *F*_ST_ (0.019) and Jost *D* (0.056) estimates between Drygały (DRY) and Jedwabno (JED) (Table 4). These two locations were also differentiated from both Augustów (AUG) and Knyszyn (KNY) whereas there was no detectable difference in the allele frequency distributions of foxes from Augustów and Knyszyn forests along the Polish border (Table 2). Łomża (LOM) and Białowieża (BIA) were not differentiated from any other location but this may be related to the small sample size. According to Structure, the most likely number of clusters in the dataset was *K* = 1 (Fig. S2), with decreasing LnPr (*D*|*K*) values and higher variability among the replicate runs at *K* > 1.

**Table 4:** Pairwise estimated values of *F*_ST_ (below diagonal) and Jost’s *D* (above diagonal) for each sampling location

	AUG	BIA	DRY	JED	KNY	LOM
AUG	–	–0.046	0.025	0.024	0.010	–0.023
BIA	–0.012	–	–0.009	–0.015	–0.034	–0.034
DRY	0.008*	–0.001	–	0.056	0.032	0.031
JED	0.008*	–0.003	0.019*	–	0.031	0.017
KNY	0.003	–0.009	0.011*	0.011*	–	0.024
LOM	–0.009	–0.011	0.011	0.006	0.008	–

*Indicates significance after Bonferroni correction

**Fig. 2:**
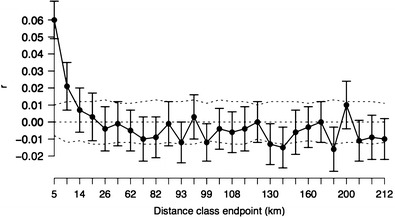
Correlogram of the average autocorrelation coefficient *r* for 28 distance classes covering the extent of the study area. The *dashed lines* represent the 95 % upper and lower bounds of the null distribution based on permutations. The *error bars* represent the 95 % confidence intervals about *r* based on bootstrapping. Significant spatial structure is observed when *r* exceeds the null distribution and the *error bars* do not overlap zero

Spatial autocorrelation among all individuals was significant (heterogeneity test *p* < 0.01) showed a pattern of decreasing relatedness with increasing distance in the first four distance classes (0 to 18 km), with an x-intercept at 21 km (Fig. 2). The *r* values were significant for the first two classes (up to 9 km) based on the permutation test, which is more conservative (Peakall et al. 2003). The approximate scale of the positive genetic structure was therefore between 9 and 21 km. The overall shape of the correlogram was similar for males and females (Fig. S3). The relationship between *r* and distance was not significantly negative until 93 km (Fig. 2).

### Landscape connectivity

The least-cost path distances are presented in Fig. 1. As expected, the Euclidean and LCP distances were highly correlated (*r* = 0.979; *p* < 0.01). There was no significant correlation between genetic distance (*F*_ST_/1 − *F*_ST_) and either Euclidean distance (*r* = −0.023; *p* = 0.49) or least-cost path distance (*r* = −0.047; *p* = 0.43) at the population level. In general, the mean resistance values for LCP lines were much lower than the resistance values for Euclidean lines (Table 5). The geographic distances along least-cost paths for LCP were much higher than for Euclidean lines, which could result in high correlation between both geographic distances (Table 5).

**Table 5:** Results of landscape connectivity analysis

	EUCL distance (km)	LCP distance (km)	Resistance for EUCL distance (cost/km)	Resistance for LCP distance (cost/km)
AUG-BIA	143	212	101.9	11.4
AUG-DRY	85	144	244.3	13.4
AUG-JED	179	254	214.9	12.9
AUG-KNY	98	145	111.3	11.5
AUG-LOM	125	183	106.9	14.0
BIA-DRY	164	225	75.9	13.8
BIA-JED	212	273	95.2	13.5
BIA-KNY	47	70	129.1	11.8
BIA-LOM	109	157	99.3	15.7
DRY-JED	96	127	173.6	13.6
DRY-KNY	135	169	116.4	14.1
DRY-LOM	82	115	101.7	15.9
JED-KNY	201	260	98.9	13.3
JED-LOM	102	128	88.5	16.4
KNY-LOM	105	138	81.3	16.2

**Fig. 3:**
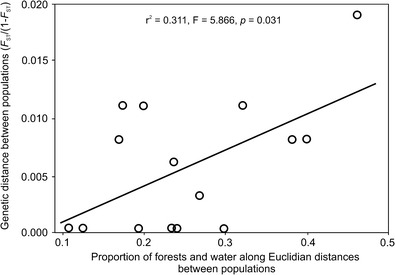
Influence of habitat structure (proportion of large forests and water) on genetic differentiation between red fox populations in north-eastern Poland

There was a significant relationship between genetic distance and the proportion of large forests along the Euclidean distances for the six red fox populations examined (*r* ^2^ = 0.272; *p* = 0.046; Table S3). Other habitats did not indicate such a correlation (*p* = 0.08–0.797; Table S3). When we investigated each of the other habitats in combination with forests, only forest edge (*r* ^2^ = 0.305, *p* = 0.033) and water (*r* ^2^ = 0.311, *p* = 0.031; Fig. 3) along the Euclidean distances showed an even stronger relationship with genetic distance. All other combinations with forests were not significant (data not shown).

## Discussion

Previous studies have successfully used microsatellite primers developed from the domestic dog genome to describe genetic diversity and structure among red fox populations (Wandeler et al. 2003; Oishi et al. 2010; Magory Cohen et al. 2012). Here, we report the first application of microsatellite loci from a domesticated red fox genome (Yan, unpublished) to natural fox populations. However, before using these loci in future studies, we recommend the redesign of primers based on DNA sequences from the study populations to avoid allele dropout due to primer binding site mismatches (Moore et al. 2010). This should reduce allele dropout and allow more loci to be retained in the final panel.

Polish red foxes harbor high genetic variation, comparable to other rural fox populations in continental Europe (Wandeler et al. 2003). Analyses of differentiation based on population level analyses (*F*_ST_ and Jost’s *D*) and Bayesian clustering revealed weak differentiation across the study area (Fig. S2; Table 4). According to the spatial autocorrelation analysis, individuals separated by up to approximately 20 km were more likely to be related to one another than expected under random mating (Fig. 2). The relationship between *r* and distance was not significantly negative until 93 km. The high heterozygosity of the population (average *H*_O_ = 0.73) is indicative of a large effective population size (Gauffre et al. 2008), which when combined with occasional long distance dispersal (juvenile dispersal of 40 km reported in Jędrzejewska and Jędrzejewski 1998) can prevent the differentiation of large populations (Wright 1943). We did not find a significant relationship between genetic and geographic (Euclidean or LCP) distances overall at the population level. It may result from continuous population distribution and occupation of all available habitats (including urban environments and large forests) (Baker et al. 2007; Janko et al. 2012). We found that it was mainly the Drygały population that was driving these patterns of an inverse relationship between genetic distance and both geographic and LCP distances. This population was the most differentiated from the other populations (Table 4), generally in spite of geographic separation (Fig. 1). When this population is removed, the relationship with genetic and geographic and LCP distances is reversed and as expected under IBD expectations (data not shown). The Drygały population is surrounded by large areas of continuous forests and lakes, habitats that are associated with high cost to movement (Fig. 1; Table 1).

Because of this, we investigated further the effects of the proportion of these habitat types between populations. We found a significant correlation between genetic distance and proportion of forests (Table S3) and a combined proportion of larger forests and both forest edge (this would be expected) and water (Table S3; Fig. 3) along the Euclidean distances for these populations and we did not find this relationship with the other habitat categories (Table S3). This was especially visible for the above-mentioned population from Drygały and Jedwabno. These types of habitat may play an important role as significant barriers for dispersal in foxes. It is consistent with radio-tracking data from Białowieża Forest (Table 1) and previous results of habitat selection analysis in Finland (Holmala and Kauhala 2009). Red foxes in Finland showed generally preferred fragmented and diverse landscape and avoided large forest patches, as well as large continuous arable fields (Holmala and Kauhala 2009). When dispersing, foxes may avoid large areas of continuous forest where food resources are less predictable. As shown by Kidawa and Kowalczyk (2011), the diet of foxes changes from *Microtus* voles to other types of food (mainly carrion) with increasing forest cover. Open or mixed habitats offer a higher abundance of food such as voles (the main food of foxes worldwide; e.g., Goszczyński 1986; Briner et al. 2007; Kidawa and Kowalczyk 2011). Food availability may shape the direction of dispersal and thus gene flow between populations, especially for less experienced juvenile foxes.

The role of water bodies and wetlands in potentially limiting dispersal in foxes is particularly apparent in Drygały and Jedwabno (Fig. 1). These populations lie in the Mazurian Lakelands and are generally significantly differentiated from the other populations (Table 4). The River Limmat was also found to influence red fox colonization in the city of Zürich, Switzerland (Wandeler et al. 2003). Population differentiation was consistently higher between foxes (in urban and rural populations) on opposite sides of the river than those on the same side. There is also indirect evidence to support the role of rivers in limiting gene flow between fox populations in Poland. Bourhy et al. (1999) described two main phylogenetic groups of the rabies virus circulating in Poland (found in foxes and raccoon dogs, *Nyctereutes procyonoides*); the Central European (CE) cluster and the North Eastern European (NEE) cluster. These two groups appear to be separated by the Vistula River, indicating limited movements of the host species across the river. Smaller rivers presumably do not impede gene flow to the same extent, especially when they are frozen in winter (Côté et al. 2012). In the future, more extensive sampling within Poland and the bordering countries should be carried out on both foxes and raccoon dogs to examine patterns of population structure as the combined population density of vector species may cross the threshold needed to sustain the virus in wildlife populations (Holmala and Kauhala 2006; Kauhala and Holmala 2006).

### Management implications

Information on the genetic structure of carnivore populations can assist the management of wildlife disease (De Young et al. 2009; Biek and Real 2010; Côté et al. 2012; Talbot et al. 2012). In this study, we observed a pattern of weak genetic structure over most of the study area. This suggests high rates of dispersal, where individuals separated by up to 20 km are genetically non-independent. De Young et al. (2009) obtained similar results for the gray fox (*Urocyon cinereoargenteus*) in an oral rabies vaccination (ORV) zone in TX, USA, where genetic data for 469 foxes at five microsatellite loci indicated local subpopulations were connected at distances greater than 30 km, beyond the established ORV zone buffer of 16–24 km (De Young et al. 2009). The management strategy in Estonia along the border with Russia is to employ a buffer zone of 30 km where natural barriers exist (lakes and large rivers) and 50 km for mainland borders where these features are absent. The country has been rabies-free for the last 4 years (Cliquet et al. 2012). Our results suggest that a similar approach would be necessary along the Polish eastern border with Belarus and Ukraine, where rabies is still common (Holmala and Kauhala 2006; Kauhala and Kowalczyk 2011; see also: http://www.who-rabies-bulletin.org).

This study is the first to characterize the genetic diversity of the red fox in north-eastern Europe and should provide a useful comparison to previous (Wandeler et al. 2003; Gachot-Neveu et al. 2009; Oishi et al. 2010; Magory Cohen et al. 2012; Statham et al. 2012) and future studies in other landscapes. Genetic diversity is high and gene flow is extensive among the fox populations in the relatively undisturbed landscape of NE Poland. We did detect local genetic structure between two populations within the Mazurian Lakelands that was likely due to the effects of large forests and water features on limiting dispersal. The subtle genetic differentiation between the Jedwabno and Drygały populations may be eroded over time, especially with the dispersal capabilities of red foxes (Wandeler et al. 2003). While more extensive sampling is required from the rest of Poland and its bordering countries, our study demonstrates that gene flow is limited primarily by geographic distance in this landscape and a vaccination buffer zone of approximately 30 km should be sufficient for the management of rabies.

## Electronic supplementary material

Below is the link to the electronic supplementary material.

ESM 1 (PDF 302 kb)
