# Supplementary material for: The influence of habitat structure on genetic differentiation in red fox populations in north-eastern Poland
Source: Acta Theriol (Warsz). 2014 Mar 22;59(3):367–76. doi: 10.1007/s13364-014-0180-2 (PMC4058057; doi:10.1007/s13364-014-0180-2)

**The influence of habitat structure on genetic differentiation in red fox populations in north-eastern Poland**

Jacinta Mullins[1], Allan D. McDevitt[1,2], Rafał Kowalczyk[1], Iwona Ruczyńska[1], Marcin Górny[1], Jan M. Wójcik[1]*

[1]Mammal Research Institute, Polish Academy of Sciences, 17-230 Białowieża, Poland

[2]School of Biology and Environmental Science, University College Dublin, Belfield, Dublin 4, Ireland

*Email: jwojcik@ibs.bialowieza.pl

## Supplementary Material

**Table S1.** Primers designed from *Vulpes vulpes* microsatellites available on GenBank

| Acc_# | motif | bp | Locus | primer | PrimerID | Sequence 5-3 | primer_Tm |
|---|---|---|---|---|---|---|---|
| JN831730.1 | (GT)19 | 123 | VVM39 | F | VVM39F | TGTCCGTCTTAAATGCAAGGAT | 61 |
| | | | VVM39 | R | VVM39R | CTTTGGAAACACCAACACACAC | 60 |
| JN831737.1 | (TG)17 | 136 | VVM246 | F | VVM246F | CATCCCCATCATATCTGGTTCT | 60 |
| | | | VVM246 | R | VVM246R | ACTCTCCAAGTCACATCCCATC | 60 |
| JN831745.1 | (GT)17 | 139 | VVM81 | F | VVM81F | GCTGAAATCCTGCTTAGAAACTTG | 61 |
| | | | VVM81 | R | VVM81R | GCTGAATGGATGAAAGGTTGAC | 61 |
| JN831734.1 | (TG)12 | 142 | VVM831 | F | VVM831F | CTGTGTTTTGTGTGTGCTGCTA | 60 |
| | | | VVM831 | R | VVM831R | GAACTGTGTACTTTCCGCTCAA | 59 |
| JN831724.1 | (AG)13 | 148 | VVM33 | F | VVM33F | CAATCAATCTGAGCACCACAAT | 60 |
| | | | VVM33 | R | VVM33R | GTAGATGAGGGGAATGTGAGGA | 60 |
| JN831726.1 | (AC)16 | 150 | VVM85 | F | VVM85F | AATTAAGTTTTCCCAGCTCCC | 59 |
| | | | VVM85 | R | VVM85R | CCTATATTGAGACCATGAGGAGG | 59 |
| JN831742.1 | (GT)11 | 165 | VVM235 | F | VVM235F | GTGTGTTCGTGTTGTTTGTGTG | 60 |

| | | | | | | | |
|---|---|---|---|---|---|---|---|
| | | | VVM235 | R | VVM235R | AATGACCTCCAAGTCTCCTGAA | 60 |
| JN831723.1 | (TG)19 | 169 | VVM219 | F | VVM219F | GTTTCTTCACAAGGGGCATAAC | 60 |
| | | | VVM219 | R | VVM219R | CACACTATTTCCATGTTTCCCA | 60 |
| JN831747.1 | (AC)18 | 177 | VVM213 | F | VVM213F | TGCAGAGACAAACCTTCAAAAC | 59 |
| | | | VVM213 | R | VVM213R | CGATCCTAAGAAACACATGGAA | 59 |
| JN831740.1 | (GT)15 | 188 | VVM529 | F | VVM529F | AAAATGACTGTGAACTGCCCTC | 61 |
| | | | VVM529 | R | VVM529R | AATGCTGAATCTTTGCTCTTCC | 60 |
| JN831731.1 | (GT)17 | 197 | VVM104 | F | VVM104F | GTGATGCAAGAAACCAAGAACA | 60 |
| | | | VVM104 | R | VVM104R | AAGAATCCAAAGGAAAAGCTCC | 60 |
| JN831735.1 | (CA)17 | 204 | VVM100 | F | VVM100F | GATACACACACACTCCCCTGTG | 60 |
| | | | VVM100 | R | VVM100R | TGCTGAAGGAAGAAAAGAGGTC | 60 |
| JN831738.1 | (GT)18 | 212 | VVM63 | F | VVM63F | AGTCCTTTGCGTGGTTCTTCT | 60 |
| | | | VVM63 | R | VVM63R | ACTGCACTCTAGCCAACTCTCC | 60 |
| JN831749.1 | (TG)17 | 226 | VVM828 | F | VVM828F | AGAAGGCACTTGTAAGGTGGAT | 59 |
| | | | VVM828 | R | VVM828R | GCACACAGACACACATGGAATA | 59 |
| JN831729.1 | (CA)14 | 234 | VVM828 | F | VVM828F | TGGAAGACCTGGAAGAGGTAGA | 60 |
| | | | VVM828 | R | VVM828R | ATTCCCTTCACCTGTATTGCTC | 59 |
| JN831739.1 | (TG)16 | 240 | VVM128 | F | VVM128F | CATTTCCTATCCTACTGCTGGC | 60 |
| | | | VVM128 | R | VVM128R | GGGAAGCCTCACATCCTTTAG | 60 |
| JN831741.1 | (GT)19 | 240 | VVM189 | F | VVM189F | GATCTGTGAGCATAAGGGTTTT | 57 |
| | | | VVM189 | R | VVM189R | TTATCCAGTCCCAAAGTCTGTC | 58 |
| JN831736.1 | (TG)17 | 244 | VVM190 | F | VVM190F | GCACATTTGAGGGTCAGTGTAA | 60 |
| | | | VVM190 | R | VVM190R | CTCCTTCCCTTCTCACCAGTAA | 60 |
| JN831748.1 | (CA)12 | 244 | VVM124 | F | VVM124F | CTCTGCTACACGGCCAAACT | 60 |
| | | | VVM124 | R | VVM124R | GGTATTCCTGTGCCTCTTGTTC | 60 |
| JN831722.1 | (TG)11 | 252 | VVM148 | F | VVM148F | GCCTAACTTCCAACCTGAAATACT | 59 |
| | | | VVM148 | R | VVM148R | TTTGTAGAGGGCATGGCAG | 60 |
| JN831744.1 | (CA)16 | 252 | VVM238 | F | VVM238F | AAATTGGAAAGTCAGCTCTTGC | 60 |
| | | | VVM238 | R | VVM238R | CAAGCAACCCATGTCAAGTAAA | 60 |
| JN831733.1 | (CA)18 | 257 | VVM25 | F | VVM25F | AAATCCTCTTATGCCTTCCGAT | 60 |
| | | | VVM25 | R | VVM25R | CCATGTTGTAGCAAATAGCAGG | 60 |
| JN831725.1 | (AC)18 | 260 | VVM812 | F | VVM812F | CTCCTTGTGGTTATGGCTGAGT | 61 |
| | | | VVM812 | R | VVM812R | TTTACCCAGAGAACGATGAAGG | 60 |
| JN831746.1 | (TG)20 | 261 | VVM844 | F | VVM844F | CTGGGTCTTGTTTTGTCTCCA | 60 |

| | | | | | | | |
|---|---|---|---|---|---|---|---|
| | | | VVM844 | R | VVM844R | GCCTCAGTAAATGCTTCCTGTT | 60 |
| JN831728.1 | (AC)15 | 264 | VVM192 | F | VVM192F | CCAGGATAATGAACAGCTTG | 56 |
| | | | VVM192 | R | VVM192R | GTTTAAGCCACCTTTAGATGAG | 55 |
| JN831727.1 | (AC)9 | 267 | VVM509 | F | VVM509F | GCATCCCTTCATTCCTGTAAAT | 59 |
| | | | VVM509 | R | VVM509R | GTGAGTGGGGAGTAGAGTGCTT | 60 |
| JN831732.1 | (TG)14 | 278 | VVM838 | F | VVM838F | AGAAATGGAAAGAGCAAGCAAG | 60 |
| | | | VVM838 | R | VVM838R | AAGAAGAGGGCAGGATGTATCA | 60 |

**Table S2.** Pilot study of 12 microsatellites in adult foxes from Augustów Forest (N = 23)

| **Locus** | ***A*** | **$H_O$** | **$H_E$** | **HWE-*p*** | **Nf** |
|---|---|---|---|---|---|
| FH2088 | 7 | 0.43 | 0.77 | < 0.01 | 0.182 |
| C466 | 6 | 0.78 | 0.79 | ns | 0 |
| FH2010 | 4 | 0.74 | 0.66 | ns | 0 |
| FH2054 | 13 | 0.83 | 0.88 | ns | 0.017 |
| C250 | 9 | 0.65 | 0.75 | ns | 0.046 |
| FH2096 | 3 | 0.43 | 0.57 | ns | 0.081 |
| FH2137 | 9 | 0.96 | 0.84 | ns | 0 |
| VVM189 | 10 | 0.96 | 0.85 | ns | 0 |
| VVM81 | 9 | 0.39 | 0.83 | < 0.01 | 0.230 |
| VVM828 | 9 | 0.78 | 0.83 | ns | 0.017 |
| VVM33 | 11 | 0.62 | 0.91 | < 0.01 | 0.278 |
| VVM124 | 7 | 0.65 | 0.69 | ns | 0.011 |

*A*: Number of alleles
$H_O$: Observed heterozygosity
$H_E$: Expected heterozygosity
HWE-p: HWE test *p*-value
Nf: Null allele frequency estimate
ns: not significant ($p > 0.05$)

**Table S3.** Proportion of different habitats on Euclidean distances (for description of habitats see Material and methods) and its influence on genetic differentiation between red fox populations in north-eastern Poland. Correlation between proportion of a given habitat and genetic distance is given at the bottom of the table

| Pairs of populations | Euclidian distance (m) | Forest | | Forest edge (300 m) | | Grasslands, wastelands, wetlands and extensive agriculture | | Villages and scattered settlements | |
|---|---|---|---|---|---|---|---|---|---|
| | | m | % | m | % | m | % | m | % |
| AUG-BIA | 142995.06 | 33428.41 | 23.38 | 26691.34 | 18.67 | 35899.20 | 25.11 | 5488.82 | 3.84 |
| AUG-DRY | 84692.39 | 21120.37 | 24.94 | 16162.45 | 19.08 | 6785.61 | 8.01 | 2275.88 | 2.69 |
| AUG-JED | 179366.83 | 55228.89 | 30.79 | 37460.63 | 20.88 | 25577.08 | 14.26 | 6543.58 | 3.65 |
| AUG-KNY | 98278.40 | 26021.94 | 26.48 | 13346.14 | 13.58 | 20177.14 | 20.53 | 7635.02 | 7.77 |
| AUG-LOM | 125187.55 | 37088.44 | 29.63 | 24615.02 | 19.66 | 35597.21 | 28.44 | 4373.77 | 3.49 |
| BIA-DRY | 163725.96 | 17453.96 | 10.66 | 27229.49 | 16.63 | 40077.20 | 24.48 | 6207.08 | 3.79 |
| BIA-JED | 211758.85 | 40704.18 | 19.22 | 45609.68 | 21.54 | 41529.51 | 19.61 | 8163.52 | 3.86 |
| BIA-KNY | 46793.29 | 11228.41 | 24.00 | 7343.23 | 15.69 | 12667.07 | 27.07 | 1389.36 | 2.97 |
| BIA-LOM | 109478.37 | 13695.39 | 12.51 | 18633.64 | 17.02 | 12062.94 | 11.02 | 5941.48 | 5.43 |
| DRY-JED | 95794.91 | 39143.42 | 40.86 | 21196.65 | 22.13 | 14694.32 | 15.34 | 1746.22 | 1.82 |
| DRY-KNY | 134933.55 | 42803.31 | 31.72 | 21102.85 | 15.64 | 33804.72 | 25.05 | 3376.29 | 2.50 |
| DRY-LOM | 81915.21 | 16353.46 | 19.96 | 16611.01 | 20.28 | 17801.83 | 21.73 | 3190.21 | 3.89 |
| JED-KNY | 201412.15 | 33167.87 | 16.47 | 38164.23 | 18.95 | 60695.13 | 30.13 | 9034.75 | 4.49 |
| JED-LOM | 102301.69 | 24252.38 | 23.71 | 19646.23 | 19.20 | 32169.03 | 31.45 | 989.09 | 0.97 |
| KNY-LOM | 104717.48 | 17699.81 | 16.90 | 16495.03 | 15.75 | 24416.36 | 23.32 | 5284.93 | 5.05 |
| **Correlation with genetic distance** | | **$r^2$=0.272; p=0.046** | | **$r^2$=0.115; p=0.217** | | **$r^2$=0.026; p=0.564** | | **$r^2$=0.112; p=0.223** | |

**Table S3. continued**

| Pairs of populations | Urban development | | Arable land | | Arable land edges (300 m) | | Water | | Forest + water |
|---|---|---|---|---|---|---|---|---|---|
| | m | % | m | % | m | % | m | % | % |
| AUG-BIA | 1803.53 | 1.26 | 9327.90 | 6.52 | 30355.86 | 21.23 | 0.00 | 0.00 | 23.377 |
| AUG-DRY | 2184.80 | 2.58 | 10016.85 | 11.83 | 14886.70 | 17.58 | 11259.73 | 13.29 | 38.233 |
| AUG-JED | 3273.28 | 1.82 | 15626.94 | 8.71 | 19140.02 | 10.67 | 16516.40 | 9.21 | 39.999 |
| AUG-KNY | 1734.82 | 1.77 | 4512.52 | 4.59 | 24584.77 | 25.02 | 266.05 | 0.27 | 26.748 |
| AUG-LOM | 0.00 | 0.00 | 5000.42 | 3.99 | 18391.26 | 14.69 | 121.43 | 0.10 | 29.723 |
| BIA-DRY | 0.00 | 0.00 | 17992.07 | 10.99 | 54766.17 | 33.45 | 0.00 | 0.00 | 10.660 |
| BIA-JED | 173.68 | 0.08 | 23147.06 | 10.93 | 52251.73 | 24.68 | 179.50 | 0.08 | 19.307 |
| BIA-KNY | 4508.02 | 9.63 | 1558.84 | 3.33 | 8098.36 | 17.31 | 0.00 | 0.00 | 23.996 |
| BIA-LOM | 424.60 | 0.39 | 21659.57 | 19.78 | 37060.74 | 33.85 | 0.00 | 0.00 | 12.510 |
| DRY-JED | 0.00 | 0.00 | 1403.01 | 1.46 | 12341.00 | 12.88 | 5270.29 | 5.50 | 46.363 |
| DRY-KNY | 0.00 | 0.00 | 4833.31 | 3.58 | 28601.12 | 21.20 | 411.96 | 0.31 | 32.027 |
| DRY-LOM | 3084.55 | 3.77 | 4081.98 | 4.98 | 20792.17 | 25.38 | 0.00 | 0.00 | 19.964 |
| JED-KNY | 8729.61 | 4.33 | 6301.02 | 3.13 | 43487.79 | 21.59 | 1831.74 | 0.91 | 17.377 |
| JED-LOM | 0.00 | 0.00 | 5975.66 | 5.84 | 19117.92 | 18.69 | 151.38 | 0.15 | 23.855 |
| KNY-LOM | 0.00 | 0.00 | 6612.91 | 6.32 | 34208.43 | 32.67 | 0.00 | 0.00 | 16.902 |
| **Correlation with genetic distance** | **$r^2$=0.;005 p=0.797** | | **$r^2$=0.217; p=0.080** | | **$r^2$=0.121; p=0.204** | | **$r^2$=0.152; p=0.150** | | **$r^2$=0.311; p=0.031** |

**Figure S1**. Statistical power of the nine microsatellite loci used in this study to detect genetic differentiation.

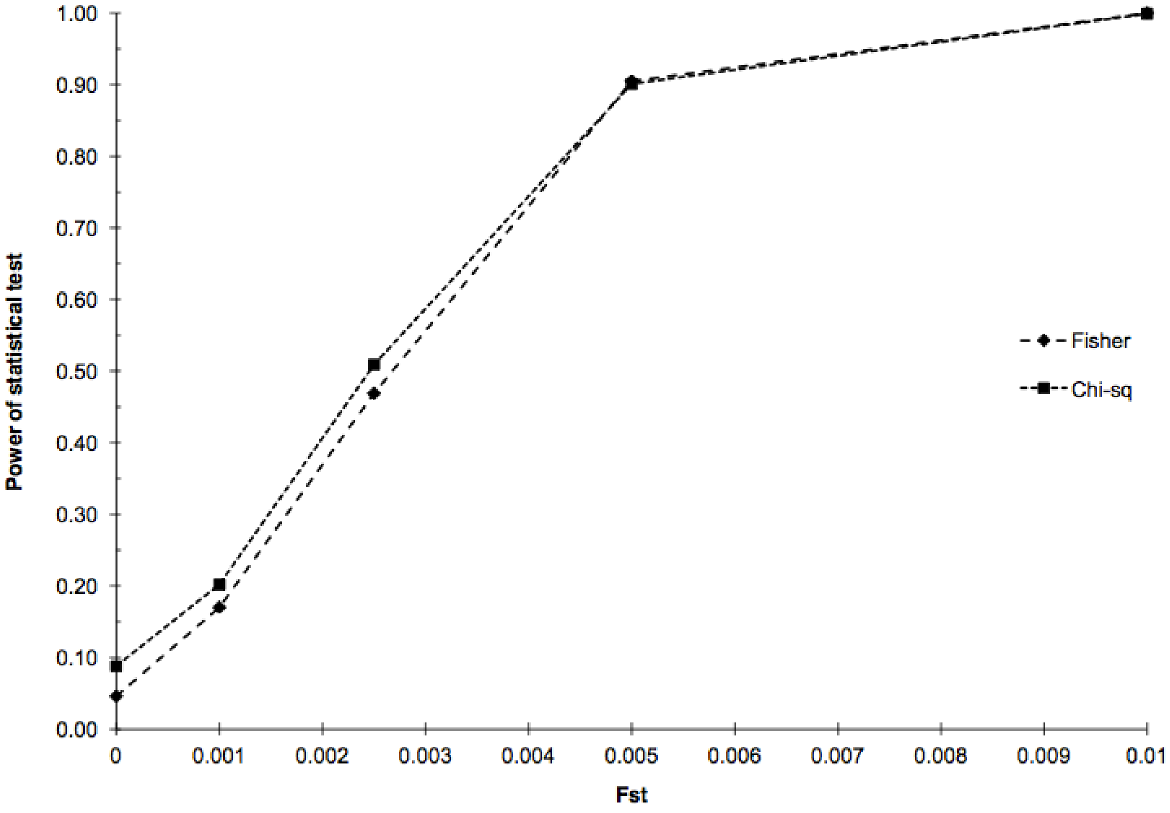

**Figure S2.** The average posterior log-likelihood probability (LnPr) of the data for each K was averaged over five replicates in STRUCTURE.

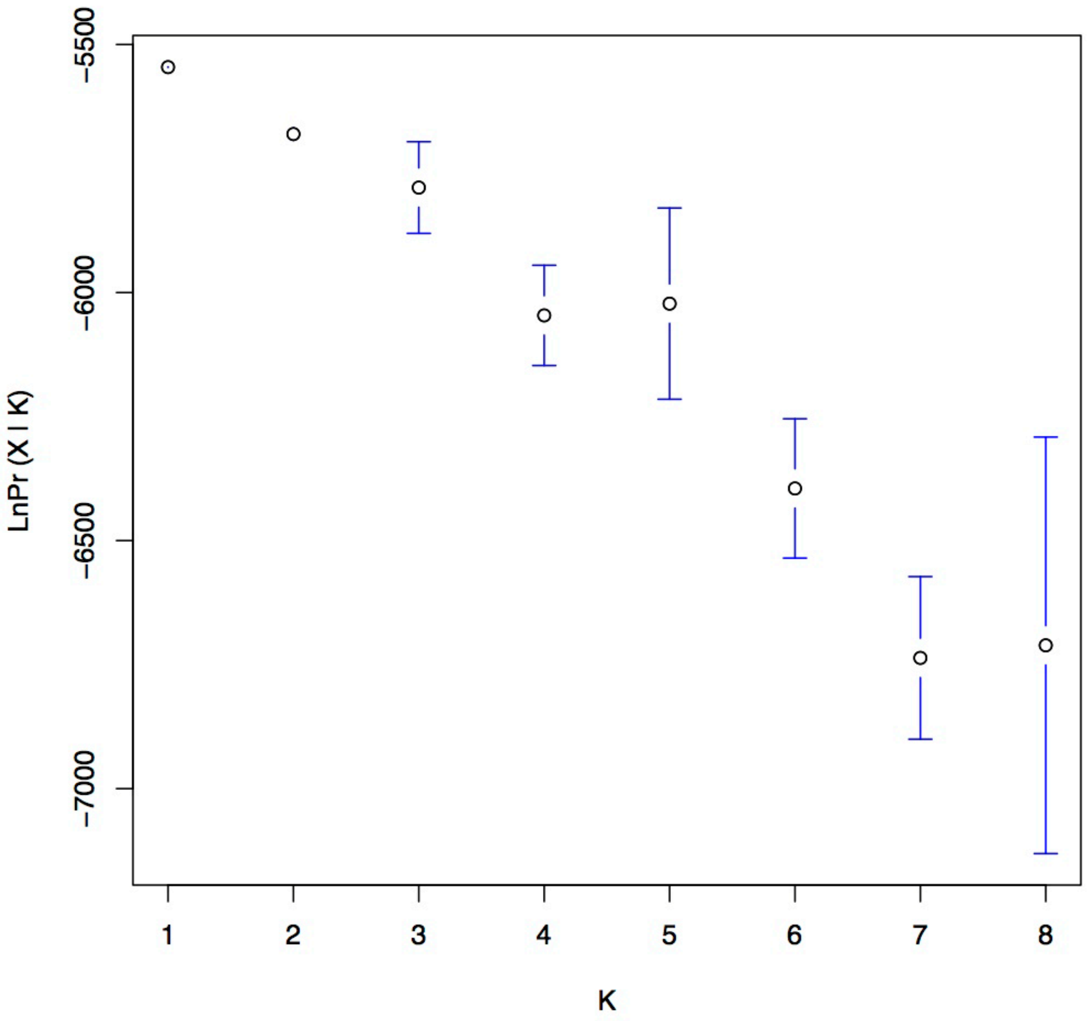

**Figure S3**. Spatial autocorrelation analysis for each sex separately. Variable distance class intervals were used to ensure sufficient sample size (>100) within each class.

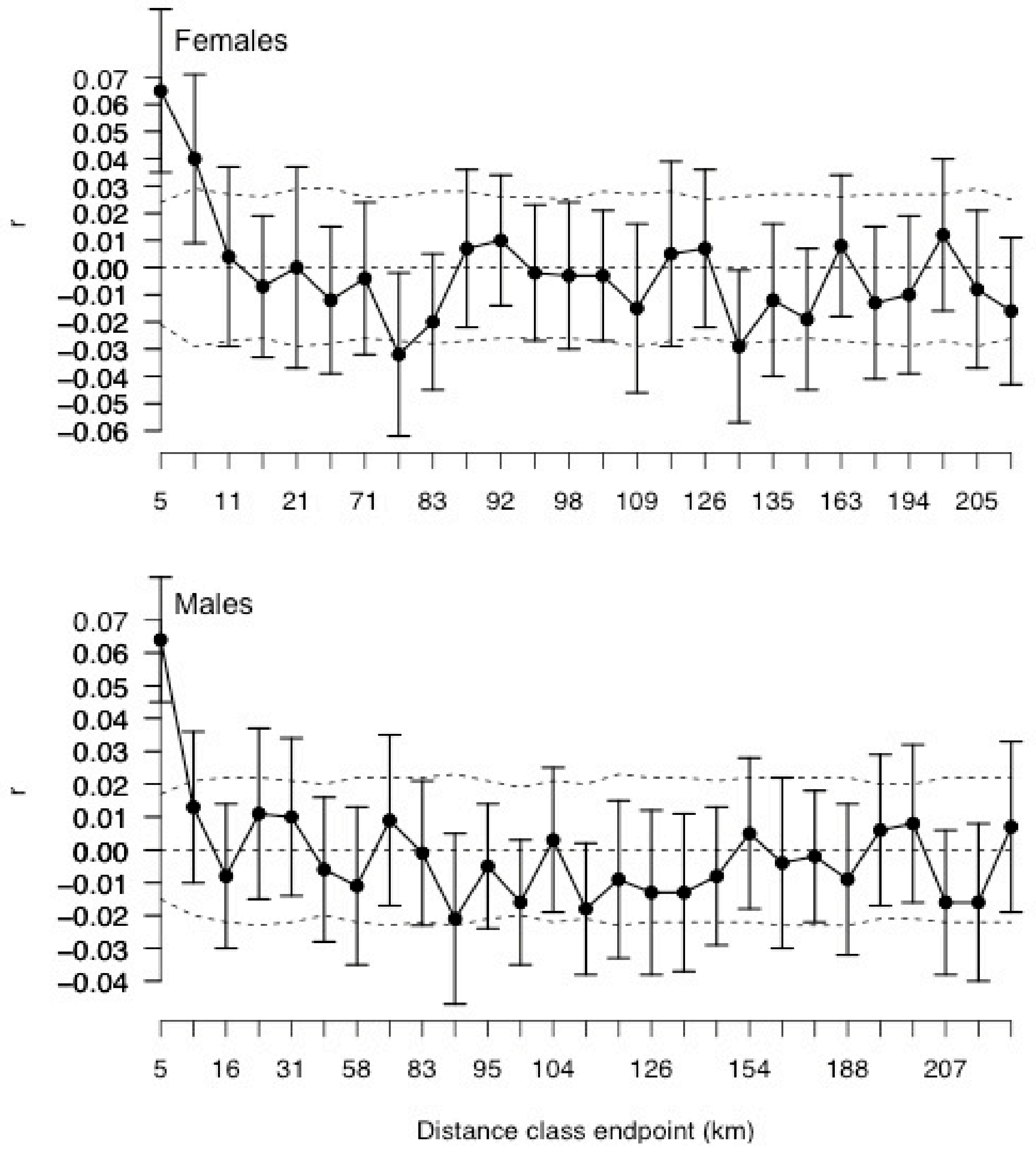

Supplement: Supplementary file 1 — (PDF 302 kb) [file 13364_2014_180_MOESM1_ESM.pdf]
